# Localization of MCT2 at peroxisomes is associated with malignant transformation in prostate cancer

**DOI:** 10.1111/jcmm.12481

**Published:** 2015-01-30

**Authors:** Isabel Valença, Nelma Pértega-Gomes, José Rámon Vizcaino, Rui M Henrique, Carlos Lopes, Fátima Baltazar, Daniela Ribeiro

**Affiliations:** a Centre for Cell Biology and Department of Biology, University of AveiroAveiro, Portugal; b Life and Health Sciences Research Institute (ICVS), School of Health Sciences, University of MinhoBraga, Portugal; c ICVS/3B's - PT Government Associate LaboratoryBraga/Guimarães, Portugal; d Department of Pathology, Centro Hospitalar do PortoPorto, Portugal; e Cancer Epigenetics Group-Research Centre, Portuguese Oncology InstitutePorto, Portugal; f Department of Pathology and Molecular Immunology, Institute of Biomedical Sciences, University of PortoPorto, Portugal; g Department of Pathology, Portuguese Oncology InstitutePorto, Portugal

**Keywords:** peroxisomes, MCT2, prostate cancer

## Abstract

Previous studies on monocarboxylate transporters expression in prostate cancer (PCa) have shown that monocarboxylate transporter 2 (MCT2) was clearly overexpressed in prostate malignant glands, pointing it out as a putative biomarker for PCa. However, its localization and possible role in PCa cells remained unclear. In this study, we demonstrate that MCT2 localizes mainly at peroxisomes in PCa cells and is able to take advantage of the peroxisomal transport machinery by interacting with Pex19. We have also shown an increase in MCT2 expression from non-malignant to malignant cells that was directly correlated with its peroxisomal localization. Upon analysis of the expression of several peroxisomal β-oxidation proteins in PIN lesions and PCa cells from a large variety of human prostate samples, we suggest that MCT2 presence at peroxisomes is related to an increase in β -oxidation levels which may be crucial for malignant transformation. Our results present novel evidence that may not only contribute to the study of PCa development mechanisms but also pinpoint novel targets for cancer therapy.

## Introduction

Metabolic adaptation is now considered a new hallmark of cancer, in which cancer cells exhibit high rates of glucose consumption and consequent lactate production 1. The crucial role of lactate exchange within the tumour microenvironment drew attention to monocarboxylate transporters (MCTs). In glycolytic tumours, they promote the efflux of lactic acid, being important players in the maintenance of the tumour intracellular pH, avoiding the routing to apoptosis and providing the favourable microenvironment conditions for invasion 2–4. MCTs have been described in a large variety of tumours and their use as targets for cancer therapy have been widely suggested. However, the importance of MCTs expression in prostate cancer (PCa) is still not well understood 5.

Prostate cancer is the second most common malignancy in men, involving challenging diagnostics 6. Several proteins, among which MCT2, have been identified as possible PCa biomarkers 7. Previous studies point out to a consistent overexpression of MCT2 in PCa cells and its possible relevance as a putative biomarker in PCa because of its high sensitivity and specificity to detect malignant glands. MCT2 protein levels were significantly up-regulated (80–100%) in PCa and prostatic intraepithelial neoplasia (PIN) lesions in human samples, in sharp contrast to the near complete lack of expression in both benign hyperplastic and normal prostate glands. These data suggest a basic mechanistic role for this protein throughout the early stages of PCa formation and prompted us to investigate this transporter in more detail 7. Similarly to MCT1, MCT2 was also found to localize in mitochondria and peroxisomes in non-tumour liver fractions 8–11. The presence of MCTs in mitochondria is justified by the need of a pyruvate carrier that plays a central role in carbohydrate and fat metabolism. In contrast, the presence of MCT1 and MCT2 at peroxisomes was explained as being involved in a lactate–pyruvate shuttle system present in the membrane of this organelle. This shuttle was suggested to play an important role in the oxidation of NADH generated by β-oxidation, being crucial for the maintenance of peroxisomal viability and, consequently, β-oxidation rates. Although the role of MCT2 in cancer is not yet elucidated, a recent study showed that MCT2 knockdown suppressed KRAS mutant (the mutation in KRAS gene occurs in a big percentage of colorectal cancers and has been suggested to be associated with proliferation and decreased apoptosis in cancer cells) colorectal tumour growth *in vivo*, supporting the use of MCT2 as a promising target for inhibition of colorectal cancer 12. However, so far, the precise localization and role of MCT2 in PCa is still unknown.

Importantly, MCT2 staining in PCa was comparable to alpha-methylacyl-CoA racemase (AMACR), an already established biomarker in PCa. Under normal physiological conditions, AMACR is expressed at appreciable levels and is transported to both the peroxisomal and mitochondrial compartments in a variety of tissues, including liver, kidney, skeletal muscle, gall bladder and brain 13–16. AMACR is responsible for the interconversion of R-configured β-methyl groups found within various small fatty acid molecules containing branched chains (such as phytols and bile acids) to the S form, a pre-requisite for metabolism *via* the β-oxidation pathway 17,18. A similar staining pattern between MCT2 and this crucial component of the oxidative metabolism raised the hypothesis that this MCT isoform might also be involved in these peroxisomal and mitochondrial-dependent mechanisms. In this work, we aimed at unravelling MCT2's intracellular localization and expression across prostate malignant transformation using different models of disease progression.

Our results demonstrate for the first time that MCT2 is present at the peroxisomes of PCa cells and that its expression increases from non-malignant to malignant cells, directly correlating with its localization at peroxisomes. Using a large series of human prostate samples, we have also shown an increase in the expression of peroxisomal β-oxidation proteins in PIN lesions and PCa cells. Our data provide novel evidence for the importance of MCT2- and peroxisomal-dependent mechanisms in PCa initiation in humans.

## Material and methods

### Cell culture

In this study, we have used several prostate cell lines such as PNT1A (non-malignant), 22Rv1 (localized tumour) and PC3 (bone metastasis). Cells were seeded in RPMI-1640 (Gibco, Invitrogen, Carlsbad, CA, USA) supplemented with 10% of foetal bovine serum (PAA Laboratories GmbH, Cölbe, Germany), 1% of antibiotic (penicillin/streptomycin) (PAA Laboratories GmbH) and incubated at 37°C in an atmosphere containing 5% CO_2_. All cell lines were cultivated under the same experimental conditions and observations were made at about 70% cell confluence.

### Antibodies and plasmids

For the immunofluorescence experiments, the following antibodies were used: MCT2 (sc-14926; Santa Cruz Biotechnology, Santa Cruz, CA, USA), MCT1 (sc-365501; Santa Cruz Biotechnology), MCT4 (sc-50329; Santa Cruz Biotechnology), CD147 (sc-71038; Santa Cruz Biotechnology), gp70 (HPA017740, 1:100; Atlas Antibodies, Stockholm, Sweden), PEX14 (a gift from Dr. Dennis Crane, Griffith University, Brisbane, Australia), catalase (ab88650; Abcam, Cambridge, UK), TRITC (Jackson ImmunoResearch, West Grove, PA, USA) and Alexa 488 (Invitrogen, Life Technologies, Carlsbad, CA, USA). For the Western blot analysis, the following antibodies were used: MCT2, ACOX1 (a gift from A. Völkl, University of Heidelberg, Germany), ACOX3 (sc-135435; Santa Cruz Biotechnology), PEX14, PMP70 (SAB4200181; Sigma-Aldrich, St Louis, MO, USA) and α-Tubulin (T9026, Sigma-Aldrich). For the immunohistochemistry (IHC) staining, the following antibodies were used: AMACR (504R-16, Cell Marque, Rocklin, CA, USA), ACOX3 (sc-135435; Santa Cruz Biotechnology) and DBP (a gift from Dr. Gabriele Moller from HelmholtzZentrum München). Pex19-YFP plasmid was a gift from Dr M. Schrader, Exeter University, UK.

### Immunofluorescence and microscopy techniques

Immunofluorescence analyses were performed in cells seeded on glass cover slips that were fixed with 4% paraformaldehyde in PBS, pH 7.4 for 20 min. Afterwards, cells were permeabilized with 0.2% Triton X-100 for 10 min., blocked with 1% BSA solution for 10 min. and incubated with primary (MCT2, MCT1, MCT4, CD147, gp70, PEX14, catalase) and secondary antibodies (TRITC or Alexa488) for 1 hr each. Between each step, cells were washed three times with PBS, pH 7.4. Lastly, cells were stained with Hoechst 33258 (PolySciences, Warrington, FL, USA) and mounted in slides using Mowiol 4-88 containing n-propylgallate. Images were obtained using a Zeiss LSM 510 Meta Confocal setup (Carl Zeiss, Jena, Germany) equipped with a plan-Apochromat 100×/1.4 oil objective. Quantifications of co-localizations were performed by determining the Manders' coefficients using the JACoP (ImageJ, Bethesda, MD, USA) software.

### Cell fractionation

22Rv1 and PC3 cells were collected in PBS with a rubber scraper. Upon centrifugation at 500 × g for 5 min., the pellet was homogenized in homogenization buffer (5 mM MOPS, pH 7.4, 250 mM sucrose, 1 mM EDTA, protease-inhibitor mixture) and passed gently through a 26-gauge syringe needle, giving rise to the total homogenate fraction (TH). A part of this homogenate was centrifuged at 1000 × g for 10 min. and the pellet corresponding to nuclei and cellular membranes were discarded. The supernatant was again centrifuged at 2500 × g for 15 min. at 4°C to separate the pellet containing heavy mitochondria. This new supernatant was centrifuged at 37,000 × g for 20 min., giving rise to a peroxisome-enriched pellet (which may also contain some degree of light mitochondria, lysosomes and endosomes) which was gently resuspended in homogenization buffer (PF). The supernatant was saved as cytosol and microsome fraction (CF). Protein concentrations of all fractions were determined by Bradford assay (Bio-Rad, Hercules, CA, USA) and 60 μg of each was loaded on the gels and subjected to Western Blot analysis.

### Western Blot

Cells were lysed with specific lysis buffer (25 mM Tris-HCl, pH 8.0, 50 mM sodium chloride, 0.5% sodium deoxycholate, 0.5% Triton X-100 and a protease-inhibitor mix). To improve protein extraction, samples were passed 20 times through a 26-gauge syringe needle and incubated on a rotary mixer at for 30 min. at 4°C. After cleared by centrifugation (17,000 × *g*, 15 min.), protein concentrations were determined by Bradford assay (Bio-Rad). Blots were incubated with the specific primary antibodies MCT2, MCT1, MCT4, Pex14, PMP70, ACOX1, ACOX3 and α-Tubulin. The antibodies were detected by a horseradish peroxidase-linked secondary antibody (HRP) using an enhanced chemiluminescence system (GE Healthcare, Waukesha, WI, USA).

### Immunoprecipitation

22Rv1 cells were transfected with Pex19-YFP using Turbofect *in vitro* transfection kit (Thermo Scientific, Marietta, OH, USA) according to the manufacturer's instructions. For immunoprecipitation of Pex19-YFP, the GFP Trap_M (Chromotek, Planegg-Martinsried, Germany) was used. Transfection with a plasmid containing GFP (GFP-C1) was used as negative control. After 48 hrs of transfection, cell pellets were incubated in lysis buffer (10 mM Tris-HCl, pH 7.5, 150 mM NaCl, 0.5 mM EDTA, 0.5% NP-40 and a protease-inhibitor mix). The lysate was cleared by centrifugation (17,000 × *g*, 15 min.) and diluted with dilution buffer (10 mM Tris-HCl, pH 7.5, 150 mM NaCl, 0.5 mM EDTA and a protease-inhibitor mix). Protein concentrations were determined by Bradford assay (Bio-Rad, Hercules, CA, USA). Ice-cold dilution buffer was used to equilibrate beads and the cell lysates were incubated for 2 hrs at 4°C on a rotary mixer. Beads were washed three times with dilution buffer and resuspended in 3× SDS sample buffer and boiled for 10 min. to elute bound proteins. Immunoprecipitated samples were separated in a 12.5% SDS-polyacrylamide gel and analysed by Western Blot.

### Patients' samples and tissue microarray construction

Prostate tissues were obtained from 480 patients with a median age of 64 years old, following radical prostatectomy. Samples, including 203 non-neoplastic, 176 high-grade PIN and 480 neoplastic tissues were used and organized into tissue microarray blocks (TMAs). The clinico-pathological data were assessed for all patients. Haematoxylin and eosin-stained sections for each tumour were examined by two independent pathologists and three 2-mm diameter representative cores from the tumour specimens were cut and placed randomly in TMA recipient blocks. Benign samples were obtained from 12 patients undergoing radical cystoprostatectomy for transitional cell carcinoma of the bladder and 10 metastatic PCa cases were obtained from clinical biopsy samples. The present study was approved by the Hospitals Local Ethical Review Committees.

### Immunohistochemistry staining and analysis

Formalin-fixed paraffin-embedded 4-μm sections were prepared from the TMA blocks. IHC technique was performed according to avidin–biotin–peroxidase complex principle [R.T.U Vectastain Elite ABC Kit (Universal), Vector Laboratories, Burlingame, CA, USA)], with the primary antibodies for AMACR (504R-16, Cell Marque), ACOX-3 (sc-135435; Santa Cruz Biotechnology) and DBP (a gift from Dr. Gabriele Moller from HelmholtzZentrum, Munich). IHC evaluation was performed blindly by two independent observers that assessed the intensity and the extension of the staining, as previously described 5.

## Results

### MCT2 localizes at peroxisomes in PCa cells

To access the exact intracellular localization of MCT2 in PCa cells, we have performed immunolocalization analyses of MCT2 in different cellular models of PCa disease progression: PNT1A (non tumour), 22Rv1 and PC3. As McClelland *et al*. (2003) have shown a peroxisomal localization of MCT2 in non-tumour liver fractions, we firstly tested whether this protein would as well be present in this organelle in PCa cells. To that end, the localization of MCT2 was analysed in parallel with the peroxisomal marker catalase.

**Fig 1:**
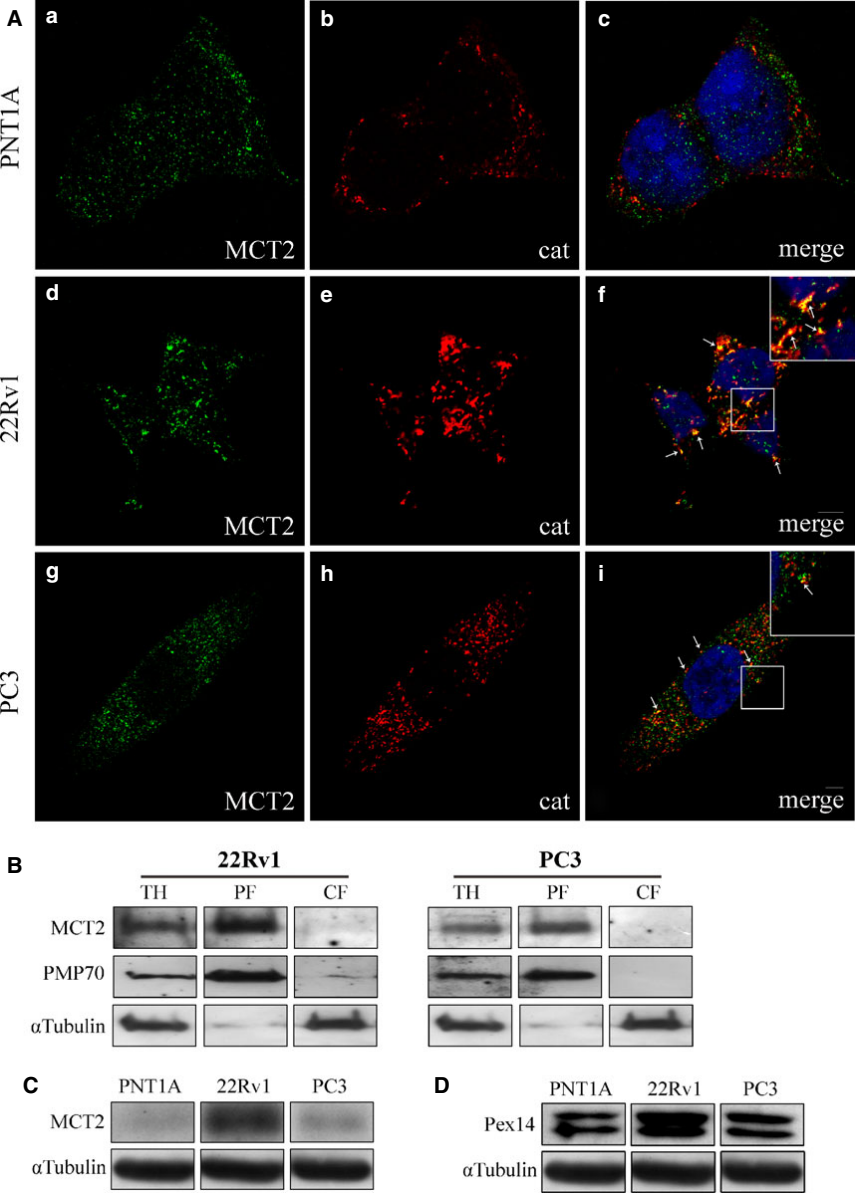
MCT2 localizes at peroxisomes in PCa cells. (A) a–c: MCT2 intracellular localization in PNT1A cells, (a) MCT2, (b) catalase and (c) merge image of a and b; d–f: MCT2 intracellular localization in 22RV1 cells, (d) MCT2, (e) catalase and (f) merge image of d and e; g–i: MCT2 intracellular localization in PC3 cells, (g) MCT2, (h) catalase and (i) merge image of g and h. Arrows indicate some of the co-localization sites. Nuclei are shown in blue (stained with Hoechst 33258). Bars represent 5 μm. (B) Western blot analysis of the presence of MCT2 in peroxisomal-enriched (PF) and cytosolic and microsomal (CF) subcellular fraction of 22Rv1 and PC3 cells upon differential centrifugation. TH represents total homogenate. PMP70 and α?-tubulin are used as peroxisome and cytosol markers, respectively. (C) Western blot analysis showing the levels of MCT2 in the different prostate cell lines models, PNT1A, 22RV1 and PC3. (D) Western blot analysis showing the levels of Pex14 in the different prostate cell lines models, PNT1A, 22RV1 and PC3.

Our results have interestingly demonstrated that, although no co-localization was observed between MCT2 and the peroxisomal marker in PNT1A, this protein is localized at peroxisomes in all the malignant cell lines (Fig.1). We have, however, observed that the localization level varied across the different models. In 22Rv1 cells, MCT2 was mainly found at peroxisomes with a minor portion spread throughout the cytoplasm as small aggregates (Fig.1A d–f). Quantification analyses show that 60.35% of the MCT2 co-localizes with the peroxisomal marker. However, the ratio of peroxisomal MCT2/cytoplasmic MCT2 decreased with disease progression, culminating with 34% of MCT2 at peroxisomes in PC3 cells (Fig.1A g–i).

To substantiate these results, we have performed differential centrifugation analyses with lysates from 22Rv1 and PC3 cells and obtained a fraction that (although presenting some degree of contamination with light mitochondria and small vesicles such as lysosomes and endosomes) is highly enriched in peroxisomes (Fig.1B, PF). The results (Fig.1B) clearly show that MCT2 localizes at the peroxisome-enriched fractions in both cell lines. Surprisingly, the amount of MCT2 at peroxisomes appears to correlate with a change on the organelle's morphology. In fact, in cells where no MCT2 was present at peroxisomes (PNT1A), this organelle exhibits a regular phenotype (with 67.7% round and 32.3% tubular; Fig.1A a–c). Curiously, in 22Rv1 cells (where MCT2 was mainly observed at peroxisomes), this organelle appears somewhat elongated and in clusters (with only 15.6% round and 84.4% tubular and in clusters). In PC3, the highly metastatic model, peroxisomes appear similar to ones in the non-malignant cells PNT1A (with 73.1% round and 26.9% tubular; Fig.1A g–i). Further experiments will have to be performed to better analyse the correlation between MCT2 localization at the peroxisomes and the different organelle morphologies.

Strikingly, the expression level of MCT2 increases from non-tumour (PNT1A) to localized malignant cells (22Rv1) in about 784% correlating to its change in localization from cytoplasmic to peroxisomal (Fig.1C). An increase in expression level (of about 89%) was also observed for Pex14 (a peroxisomal membrane protein; Fig.1D), suggesting an increase in peroxisomal membrane surface/number accompanying the malignant transformation.

### Other MCT isoforms are present at the peroxisomes, plasma membrane, cytoplasm and nucleus of PCa cells

As McClelland *et al*. (2003) have also shown a peroxisomal localization of MCT1 in non-tumour liver fractions, we decided to test whether this protein would also be present in this organelle in PCa cells.

**Fig 2:**
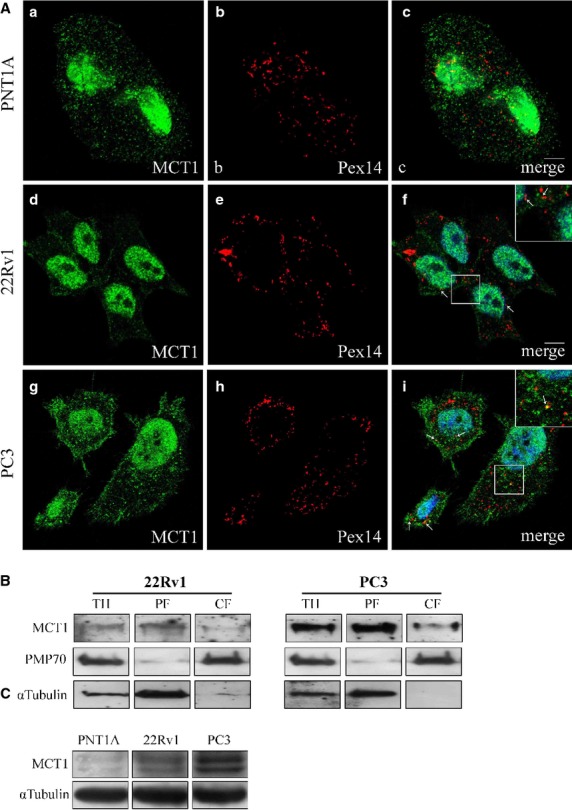
MCT1 localizes at peroxisomes, cytoplasm, nucleus and plasma membrane in PCa cells. (A) a–c: MCT1 intracellular localization in PNT1A cells, (a) MCT1, (b) Pex14 and (c) merge image of a and b; d–f: MCT1 intracellular localization in 22RV1 cells, (d) MCT1, (e) Pex14 and (f) merge image of d and e; g–i: MCT1 intracellular localization in PC3 cells, (g) MCT1, (h) Pex14 and (i) merge image of g and h. Arrows indicate some of the co-localization sites. Nuclei are shown in blue (stained with Hoechst 33258). Bars represent 5 μm. (B) Western blot analysis of the presence of MCT1 in peroxisomal-enriched (PF) and cytosolic and microsomal (CF) subcellular fraction of 22Rv1 and PC3 cells upon differential centrifugation. TH represents total homogenate. PMP70 and α-tubulin are used as peroxisome and cytosol markers, respectively. (C) Western blot analysis showing the levels of MCT1 in the different prostate cell lines models, PNT1A, 22RV1 and PC3.

Upon immunolocalization of MCT1 together with peroxisomal markers, we have observed a small degree of co-localization with the peroxisomal marker (Fig.2A) in the tumour cell lines (5.15% of the MCT2 co-localizes with the peroxisomal marker in 22RV1 cells and 4% in PC3 cells). Differential centrifugation analyses with lysates from 22Rv1 and PC3 cells (Fig.2B) clearly confirm that MCT1 localizes at the peroxisome-enriched fractions in both cell lines.

However, MCT1 was mainly found to strongly localize at the nucleus in all cell lines (Fig.2A) and was also present at the cytoplasm and plasma membrane (Fig.2A). The expression level of MCT1 increased from the PNT1A cells to the tumour cell lines, with a higher expression at PC3 cells then at 22RV1 cells (Fig.2C).

**Fig 3:**
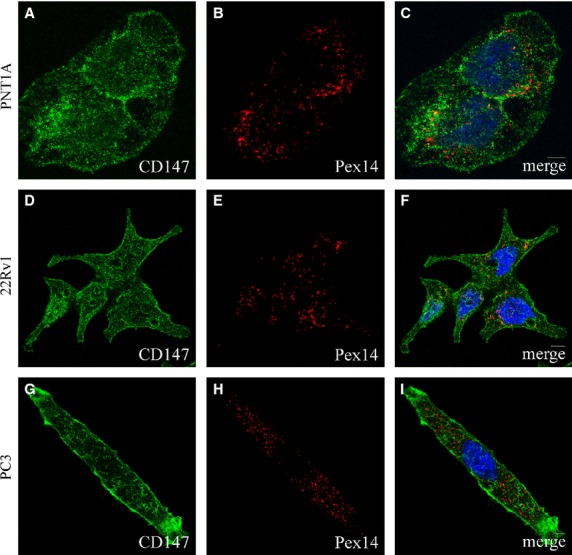
CD147 localizes at the cytoplasm, nuclear and plasma membranes in PCa cells. A–C: CD147 intracellular localization in PNT1A cells, (A) CD147, (B) Pex14 and (C) merge image of A and B; D–F: CD147 intracellular localization in 22RV1 cells, (D) CD147, (E) Pex14 and (f) merge image of D and E; G–I: CD147 intracellular localization in PC3 cells, (G) CD147, (H) Pex14 and (I) merge image of G and H. Nuclei are shown in blue (stained with Hoechst 33258). Bars represent 5 μm.

Interestingly, MCT1 chaperone CD147 was found to localize not only at the plasma membrane and cytoplasm but also at the nuclear envelope, mainly in 22RV1 cells (Fig.3).

**Fig 4:**
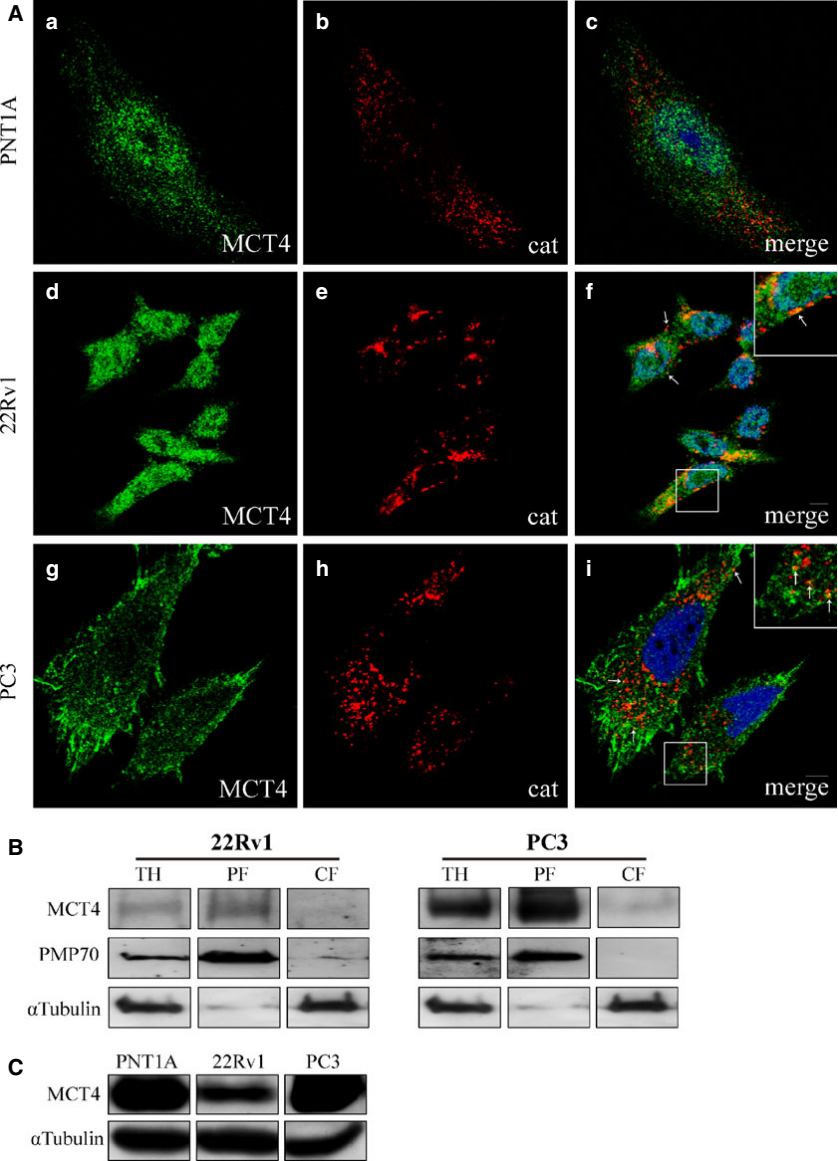
MCT4 localizes at peroxisomes, cytoplasm and plasma membrane in PCa cells. (A) a–c: MCT4 intracellular localization in PNT1A cells, (a) MCT4, (b) catalase and (c) merge image of a and b; d–f: MCT4 intracellular localization in 22RV1 cells, (d) MCT4, (e) catalase and (f) merge image of d and e; g–i: MCT4 intracellular localization in PC3 cells, (g) MCT4, (h) catalase and (i) merge image of a and g and h. Arrows indicate some of the co-localization sites. Nuclei are shown in blue (stained with Hoechst 33258). Bars represent 5 μm. (B) Western blot analysis of the presence of MCT4 in peroxisomal-enriched (PF) and cytosolic and microsomal (CF) subcellular fraction of 22Rv1 and PC3 cells upon differential centrifugation. TH represents total homogenate. PMP70 and α-tubulin are used as peroxisome and cytosol markers, respectively. (C) Western blot analysis showing the levels of MCT4 in the different prostate cell lines models, PNT1A, 22RV1 and PC3.

As the intracellular localization of MCT4 in PCa cells has never been assessed, we have also analysed it by immunolocalization with organelle markers. MCT4 was found mainly at the cytoplasm in all the three cell lines with some degree of localization at the peroxisomes (6.78% of the MCT4 co-localizes with the peroxisomal marker in 22RV1 cells and 4.77% in PC3 cells). In PC3 cells, however, a strong plasma membrane staining was also observed (Fig.4A). The peroxisomal localization in both 22Rv1 and PC3 cells was confirmed by differential centrifugation analyses (Fig.4B). The expression level of MCT4 is similar in PNT1A and PC3 cells, decreasing in 22Rv1 cells (Fig.4C).

### MCT2 travels to the peroxisomal membranes *via* interaction with PEX19

The quick movement of monocarboxylates across the membranes is imperative for cellular metabolism. These proteins are thought to require chaperones such as CD147 in the case of MCT1 and MCT4, or gp70 in the case of MCT2, for appropriate expression and activity in the plasma membrane. As MCT2 was the main isoform found to strongly localize at peroxisomes in PCa cells, we aimed at better unravelling its targeting mechanism to this organelle. A previous study was unable to find gp70 expression in human prostate samples that exhibited MCT2 expression 5. However, our studies with confocal microscopy allowed us to observe some, although scarce, gp70 distributed in the cytoplasm without any co-localization with peroxisomal markers (results not shown). Hence, the protein that actually behaves as chaperone for MCT2's transport to peroxisomes in prostate cells remained unknown.

**Fig 5:**
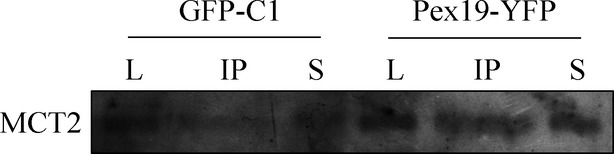
MCT2 interacts with Pex19. Co-immunoprecipitation analysis of the Interaction between endogenous MCT2 and overexpressed Pex19 (Pex19-YFP) in 22RV1 cells. Negative controls were performed by immunoprecipitating cells expressing GFP (GFP-C1). Western blots were performed with an antibody anti-MCT2 as well as tubulin as loading control. L represents lysate, IP represents the immunoprecipitation result and S represents the supernatant.

As Pex19 is the main responsible for the transport of peroxisomal membrane proteins to this organelle 19,20, we tested whether this protein could act as a chaperone for MCT2 in these cells. As in 22RV1, the majority MCT2 was present at peroxisomes, it was the chosen model to study the possible interaction between MCT2 and PEX19. 22RV1 cells transfected with Pex19-YFP were then subject to immunoprecipitation experiments. Our results showed, indeed, an interaction between MCT2 and PEX19 (Fig.5), suggesting that MCT2 is able to take advantage of peroxisomal transport machinery to reach this organelle.

### The expression of MCT2 and peroxisomal β-oxidation-related proteins increase in prostate malignant transformation

**Fig 6:**
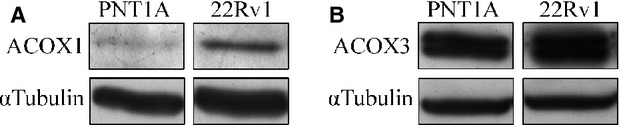
Peroxisomal β-oxidation proteins are overexpressed in localized prostate tumour cells. (A) Western blot analysis, showing the expression levels of ACOX1 in 22RV1 cells. (B) Western blot analysis, showing the expression levels of ACOX3 in 22 RV1 cells.

McClelland *et al*. (2003) observed a decrease in β-oxidation upon MCTs inhibition, suggesting that the presence of MCT2 at peroxisomes of non-malignant liver cells would be essential for the maintenance of peroxisomal viability and consequently β-oxidation rates. Hence, we hypothesized that the increase in MCT2 expression from non-malignant prostate cells (PNT1A) to localized prostate tumour cells (22RV1) could be related with an increase in peroxisomal β-oxidation. In fact, we observed an increase in the expression levels of ACOX1 (of about 246%; Fig.6A) and ACOX3 (about 14%; Fig.6B), two central proteins in the peroxisomal β-oxidation pathway 18,21,22. These results interestingly suggest that, indeed, the increase in MCT2 expression levels as well as its presence at peroxisomes, are related to an increase in β-oxidation levels which may be crucial for malignant transformation. Further experiments need to be performed to confirm this correlation.

### Peroxisome-related proteins are overexpressed in human PCa samples

**Fig 7:**
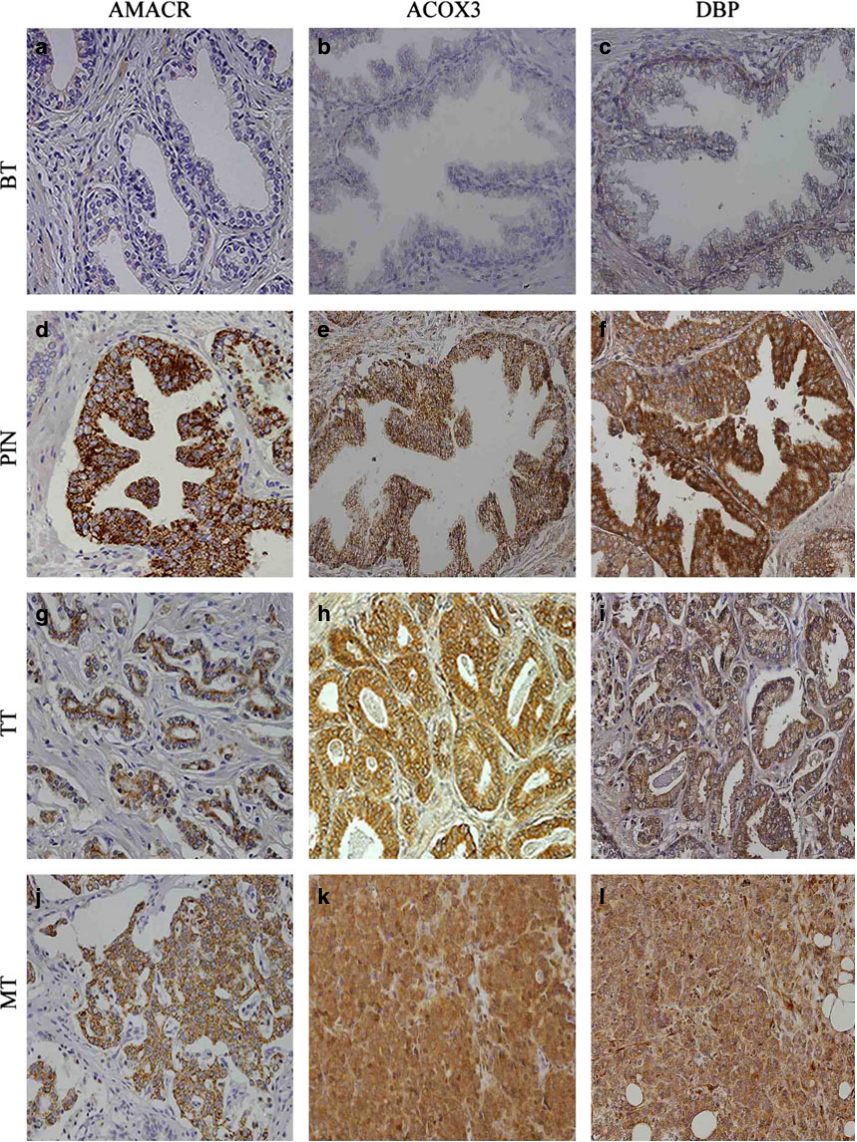
The expression of proteins involved in peroxisomal β-oxidation is more intense in PIN lesions and prostate tumour samples. Immunohistochemical expression of metabolic-related proteins in benign tissue (BT), PIN lesions (PIN), prostate tumour tissue (TT) and metastatic tissue (MT; 200× magnification). Images are shown with a magnification of 200×.

**Fig 8:**
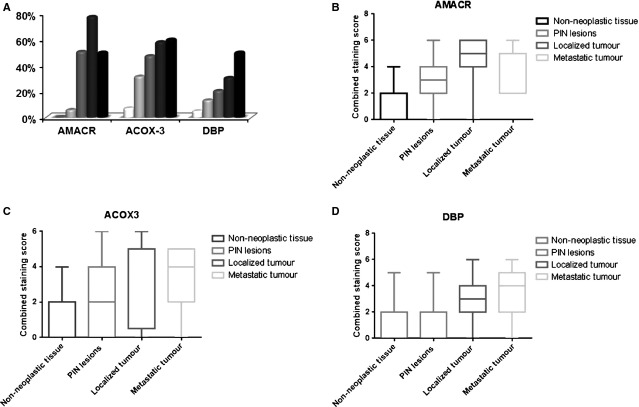
The percentage of cases showing increase AMACR, ACOX3 and DBP expression increases in PIN lesions and tumour samples. (A) Overall percentage of positive cases for each one of the proteins studied in the different tissue samples. (B–D) Distribution of the final staining score for AMACR (B), ACOX3 (C) and DBP (D) in different PCa tissues.

To study the pathological relevance of the expression of proteins involved in peroxisomal β-oxidation in human samples, we characterized the expression of AMACR, ACOX3 and DBP in a large series of human prostate samples. Figure7 shows representative immunohistochemical reactions for all proteins in BT (benign tissue), PIN lesions, primary tumour tissue (TT) and metastatic tissue (MT). We observed important changes in the expression of all the proteins studied from benign and/or adjacent non-neoplastic prostate tissue to PIN lesions and to primary tumour. AMACR, ACOX3 and DBP expressions was clearly increased from BT to TT. Figure8A shows the specific percentage of positive cases for each protein in different tissues. Interestingly, an evident increase was verified from BT or NNT to PIN and TT. In Figure8B and C, the distribution of the final score across different prostate tissue types for each protein is represented, showing in a general way that there is an increase in the final score in the localized tumour when compared to the benign glands.

## Discussion

A variety of studies pointed to the importance of MCTs in solid tumours. In contrast to MCT1 and MCT4, which were mainly described at the plasma membrane in a wide variety of malignancies, MCT2 expression in human cancers was always less evident and, when present, its expression was mainly cytoplasmic 23. As so, and because of the major role described for MCTs as important players in the acid-resistant phenotype of tumour cells, MCT1 and MCT4 turned into the most popular isoforms explored for cancer therapy. However, a study in malignant gliomas and more recently a study in colorectal malignancies showed a significant role for MCT2 in cancer, describing that MCT2 inhibition induces senescence-associated mitochondrial dysfunction and suppresses progression of colorectal malignancies *in vivo* 12,24. Similarly to what was described for colon cancer, MCT2 expression in PCa was also observed in the cytoplasm of tumour cells 7. The observation that MCT2 was clearly expressed in PIN lesions and prostate tumour cells strongly pointed into an unexplored role of this isoform in prostate malignant transformation.

In this study, we have demonstrated for the first time that MCT2 is localized at peroxisomes in PCa cells. Importantly, its localization pattern changes across the different *in vitro* models of prostate disease progression: while no peroxisomal localization was observed in non-malignant prostate cells, the highest co-localization level was detected in the localized tumour cells, decreasing with the level of metastization. These results strongly suggest that the localization of MCT2 in PCa peroxisomes is important in disease initiation.

Peroxisomes are ubiquitous and essential subcellular organelles, versatile and highly diverse depending on the organism, cell type and developmental stage 22,25–27. They fulfil important functions in lipid and reactive oxygen species metabolism, influencing, among others, neuronal development and ageing 22,25–27. The protein composition, morphology and abundance of these dynamic organelles are tightly regulated upon external stimuli to maintain cellular homoeostasis 22,25–27. Peroxisome dynamics and morphology play important roles in cell pathology, and defects on these machineries lead to significant implications in health and disease 26. Information on the role of peroxisomes in tumour development is scarce. In some studies, mainly on hepatocarcinomas, a decrease in peroxisome number in cancer cells was demonstrated by the reduction in peroxisomal catalase and the three peroxisomal β-oxidation enzymes 28.

The presence of MCT2 in peroxisomes (in non-tumour liver cells) was firstly suggested by McClelland *et al*. (2003) who proposed that this protein would be involved in a redox shuttle system at the peroxisomal membrane, consisting of a substrate cycle between lactate and pyruvate. This shuttle would stimulate the reoxidation of NADH, fuelling the organelles very long-chain fatty acid β-oxidation and playing a role in peroxisomal redox balance (McClelland *et al*., 2003). Our results seem to highlight a similar role for MCT2 at the peroxisomal membrane of PCa cells. In parallel to a clear increase on MCT2 expression from non-tumour to localized tumour cells, we have also observed a rise in the expression of specific key proteins involved in peroxisomal β-oxidation: ACOX1 and ACOX3. These results suggest that the presence of MCT2 at peroxisomes stimulates an increase in the β-oxidation rate that seems to be related with prostate tumour initiation. Importantly, these results are substantiated by the study of the expression of AMACR, ACOX3 and DBP in human prostate samples, showing a specific and consistent overexpression of proteins involved in peroxisomal fatty acid oxidation in PCa as well as in PIN lesions in contrast to benign tissue, suggesting a possible aetiological role for this pathway in malignant transformation.

The observation that the expression level of MCT2 as well as its co-localization with peroxisomes decreases from the localized tumour cells to the highly metastatic models likely demonstrates that in these cells other metabolic mechanisms play a more important role, such as hypoxia and hypoxic-related proteins involved in glycolysis, which was already suggested to be linked with disease aggressiveness 29. To better unravel the mechanisms involved in the peroxisomal and MCT2-dependent disease initiation, we have analysed the cellular trafficking of MCT2 and demonstrated that GP70, the previously described MCT2 chaperone, is barely expressed in the PCa cells, indicating that MCT2 should rely on an alternative chaperone for its proper function in these cells. Our results show that MCT2 interacts with Pex19, the main responsible for the trafficking of intrinsic peroxisomal membrane proteins to this organelle. These data strongly suggest a highjack of the peroxisomal transport machinery to sustain malignant transformation. We have furthermore observed a clear change in peroxisome morphology across prostate malignant transformation correlated with MCT2 presence at this organelle, providing once more evidence for the involvement of these organelles in tumour initiation and progression.

In this study, we have also analysed the intracellular localization of MCT1 and MCT4 in PCa cells. Surprisingly, both proteins are also found at peroxisomes, although in a much lower extent than MCT2. It is tempting to suggest that both MCT1 and MCT4 would be present at the peroxisomal membranes as a partner for MCT2 within the substrate shuttle. In fact, McCleeland *et al*. (2003) have already shown MCT1 to be present in liver peroxisomes and to form, along with peroxisomal lactate dehydrogenase, a peroxisomal lactate shuttle. However, our results show that the amount of MCT1 at peroxisomes is much lower than the one of MCT2. Furthermore, MCT1 is also present at the plasma membrane and, surprisingly, at the nucleus. Its chaperone CD147 was found to localize at the nuclear membrane, suggesting that it was the responsible for the MCT1 transport to the nuclear membrane prior to its internalization. A nuclear localization for MCT1 has already been shown to occur in human sarcomas 30. Also, only a small part of MCT4 was found at peroxisomes with its majority localizing in the cytoplasm and at the plasma membrane, as expected. Although the possibility of MCT1 and/or MCT4 behaving as partners of MCT2 at the peroxisomal membrane is very appealing, further experiments have to be performed to test this or other hypothesis concerning their role at this organelle. Nevertheless, our results suggest that the presence of multiple MCTs is physiologically important in cancer cells and the involvement of these isoforms in the biology of tumour cells goes beyond their classical role in the glycolytic metabolism.

Our study describes for the first time the presence of MCT2 at the peroxisomes of PCa cells and suggests a possible role for peroxisome-related mechanisms in prostate malignant transformation. These results may further be exploited for the study of peroxisomal metabolism as target for cancer therapy.

## Supporting Information

**Figure S1** MCT2 localizes at peroxisomes in PCa cells.

**Figure S2** MCT1 localizes at peroxisomes in PCa cells.

**Figure S3** CD147 localizes at the cytoplasm, nuclear and plasma membranes in 22RV1 cells.

**Figure S4** MCT4 localizes at peroxisomes in PCa cells.
