# Supplementary figures and images for: Localization of MCT2 at peroxisomes is associated with malignant transformation in prostate cancer
Source: J Cell Mol Med. 2015 Jan 30;19(4):723–33. doi: 10.1111/jcmm.12481 (PMC4395187; doi:10.1111/jcmm.12481)

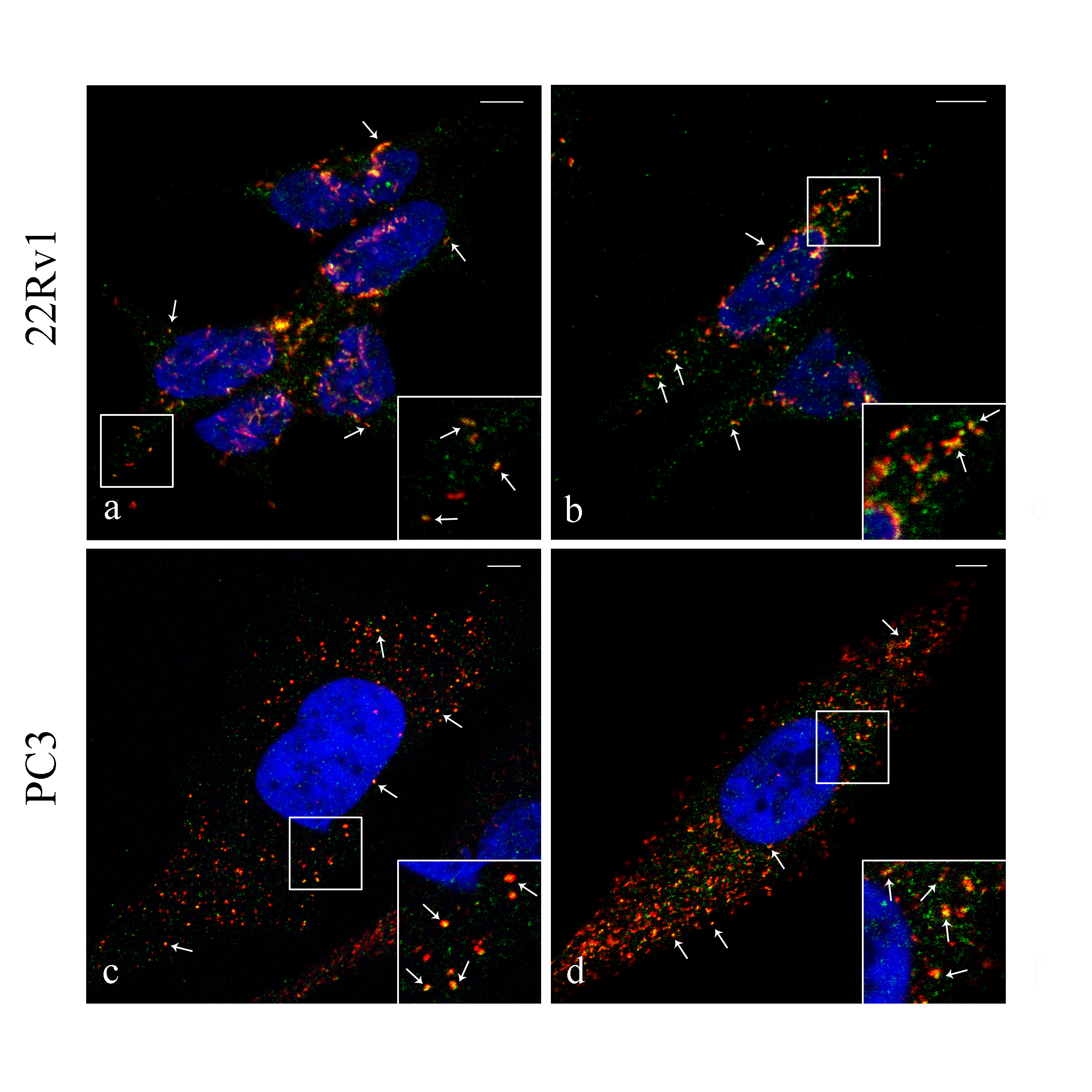

Supplement: Supplementary file 1 [file jcmm0019-0723-sd1.tif]

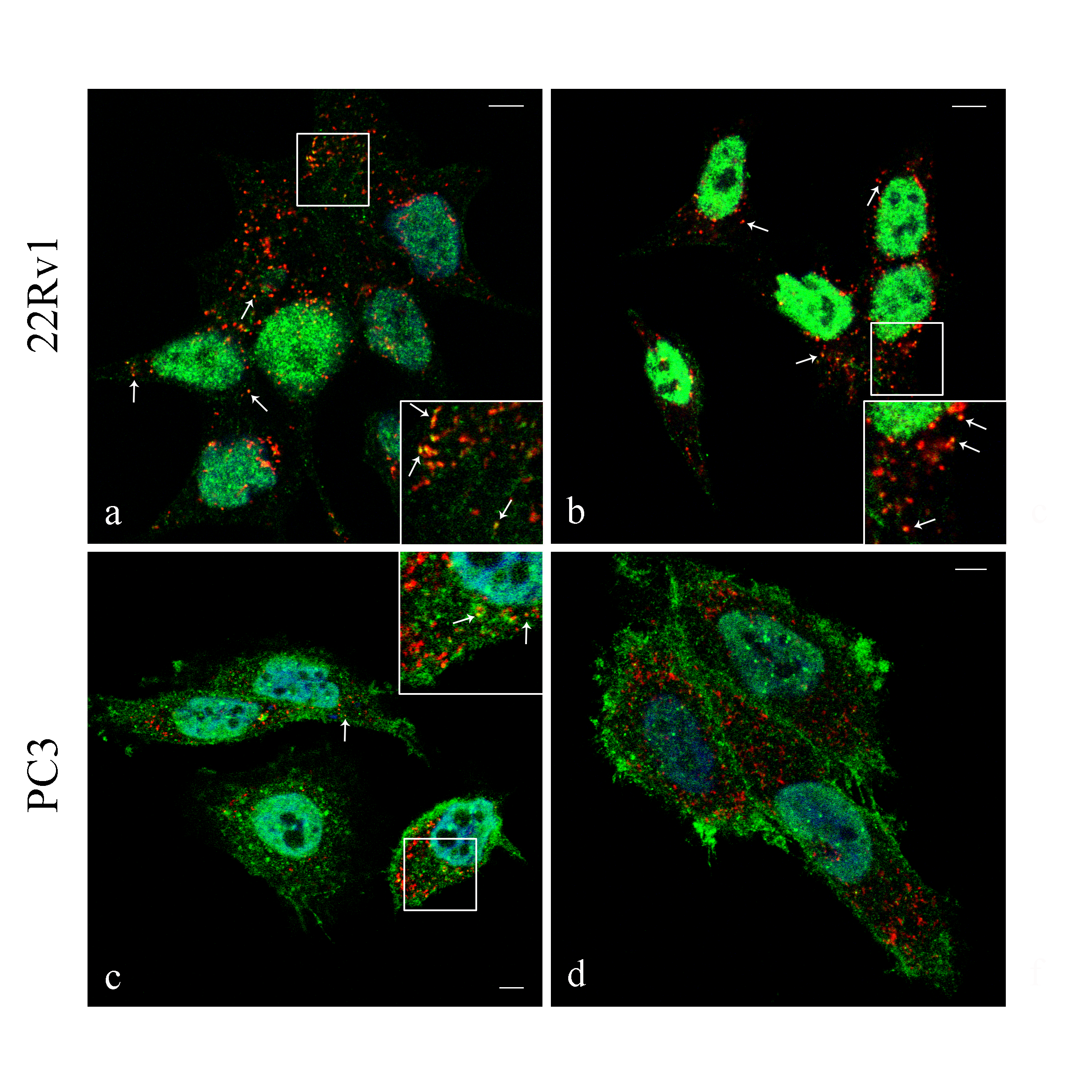

Supplement: Supplementary file 2 [file jcmm0019-0723-sd2.tif]

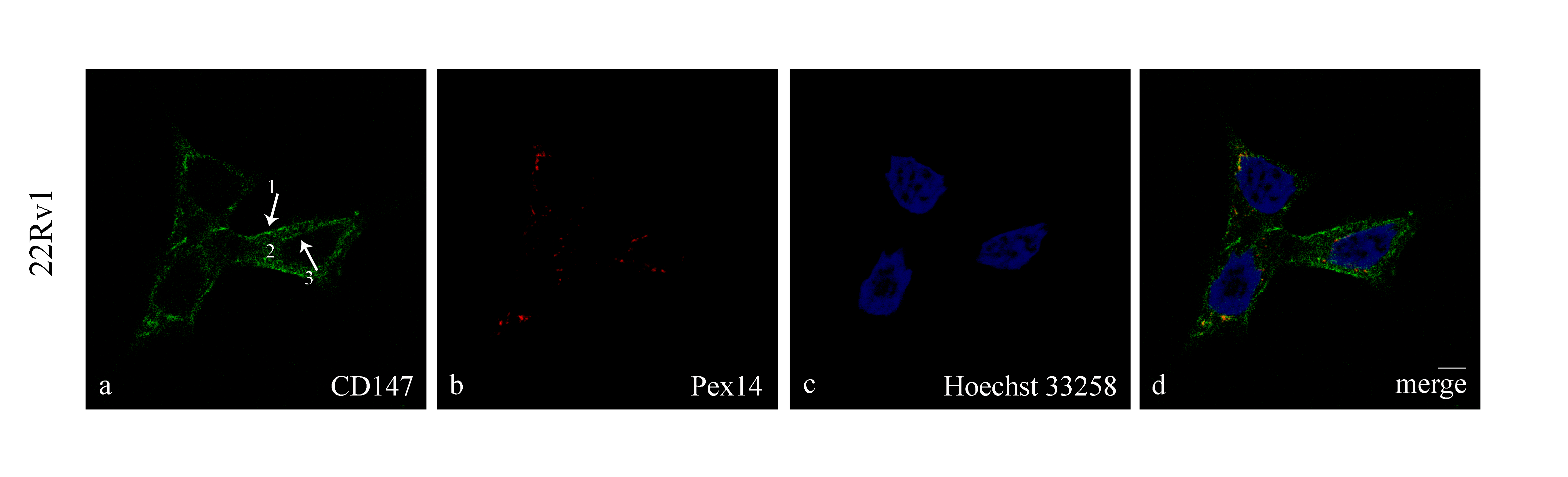

Supplement: Supplementary file 3 [file jcmm0019-0723-sd3.tif]

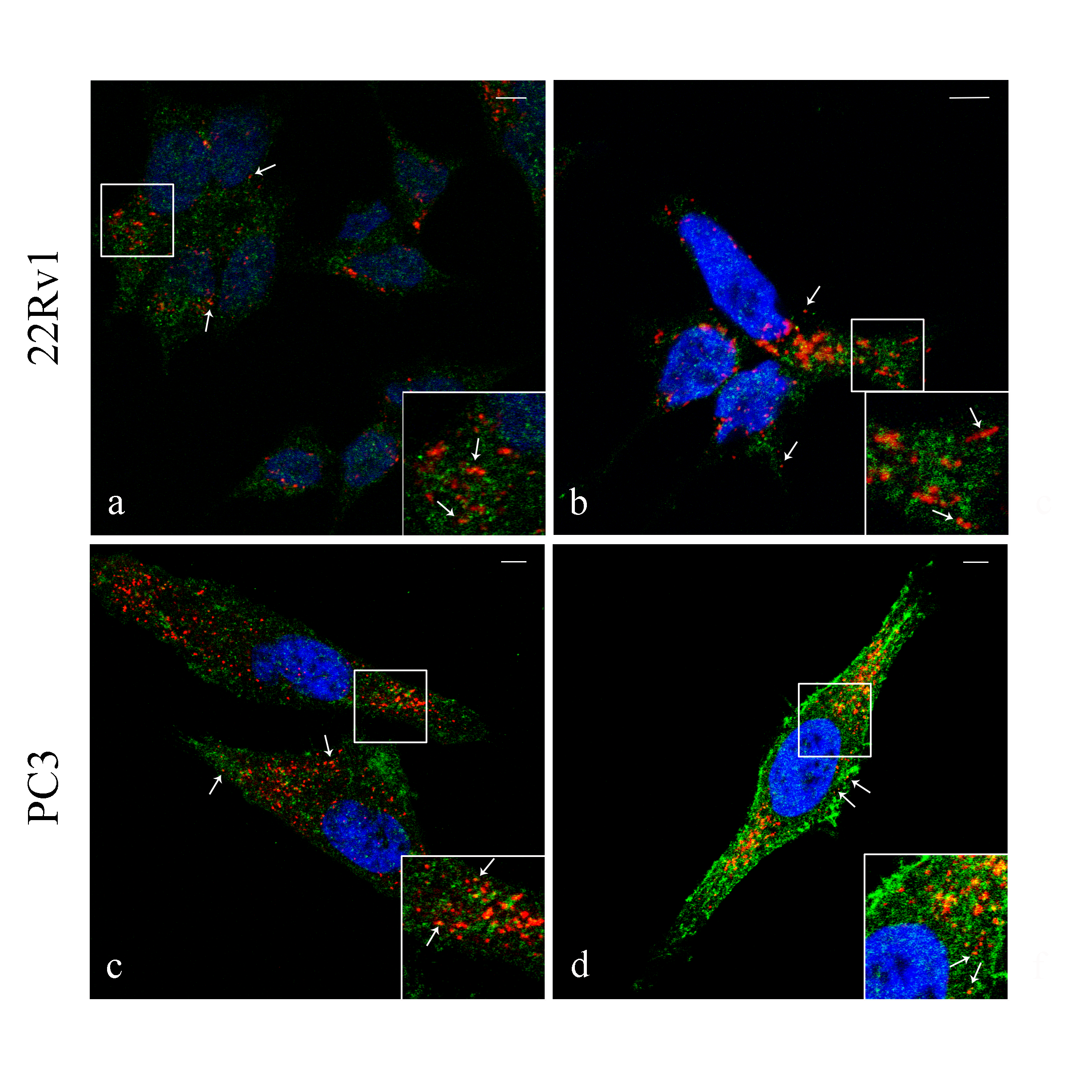

Supplement: Supplementary file 4 [file jcmm0019-0723-sd4.tif]
